# Factors Influencing HPV Vaccine Intentions in Malaysian Men Who Have Sex with Men: A Cross-Sectional Study in Malaysia

**DOI:** 10.3390/pathogens12101261

**Published:** 2023-10-19

**Authors:** Li Ping Wong, Haridah Alias, Sin How Lim

**Affiliations:** 1 Centre for Epidemiology and Evidence-Based Practice, Department of Social and Preventive Medicine, Faculty of Medicine, Universiti Malaya, Kuala Lumpur 50603, Malaysia; haridahalias@gmail.com; 2 Department of Social and Preventive Medicine, Faculty of Medicine, Universiti Malaya, Kuala Lumpur 50603, Malaysia; 3 Centre of Excellence for Research in AIDS (CERiA), Faculty of Medicine, Universiti Malaya, Kuala Lumpur 50603, Malaysia

**Keywords:** HPV vaccination, homosexual, marginalized population

## Abstract

In the landscape of healthcare disparities and the marginalized status of men who have sex with men (MSM) in Malaysia, understanding the dynamics surrounding HPV vaccination is of paramount importance. The purpose of this study is to examine the knowledge and attitudes of MSM regarding HPV vaccination and to identify factors that may hinder or facilitate its uptake. The findings will contribute to the development of targeted interventions to promote HPV vaccination and reduce the burden of HPV-related health issues among Malaysian MSM. Between May 2019 and September 2022, an online cross-sectional survey was conducted to collect data through popular social media platforms targeting MSM in Malaysia. A multivariable logistic regression model was employed to investigate the associations between HPV vaccination intention and various influencing factors. Out of the total 411 respondents in the study, 266 (60.3%) indicated an intent to receive the HPV vaccination, falling under the categories of “certain to happen”, “very likely”, and “likely”. The average knowledge score for participants was 6.82 (SD = 3.93, range 0–13) out of a total possible score of 13. In the multivariate logistic model, participants who identified themselves as bisexual (OR 6.93, 95% CI 2.35–20.41) and gay/homosexual (OR 4.36, 95% CI 1.66–11.42) showed a greater inclination to receive the HPV vaccine compared to heterosexual participants. High intent to be vaccinated for HPV infection was positively and significantly associated with a high level of knowledge (OR 1.79, 95% CI 1.09–2.95). In the multivariable model, there was no significant association between all variables of attitudes towards HPV infection and HPV vaccinations and the intention to receive HPV vaccination. Study participants reported a low level of susceptibility to HPV infection despite their perception that HPV infection is severe. Two-thirds of participants expected to encounter stigma in healthcare settings during future implementation of HPV vaccination programs. This study underscores the importance of improving HPV vaccine acceptance among Malaysian MSM due to the moderate acceptance level observed. In Malaysia, promoting HPV awareness, enhancing risk perception, and addressing stigma and sensitivity surrounding HPV vaccination may be beneficial in increasing the vaccination willingness among MSM.

## 1. Introduction

The Human Papillomavirus (HPV) is a sexually transmitted infection (STI) affecting both men and women. Infection with HPV is widely recognized as a significant risk factor for the development of anal cancer [1]. According to the American Cancer Society, there were an estimated 9090 new cases of anal cancer and 1430 deaths attributable to anal cancer in 2021 alone [2]. Globally, the incidence of anal cancer has increased significantly, with approximately 20,000 new cases being reported each year [3]. Additionally, infection with HPV is acknowledged as a major risk factor for the development of penile cancer. According to a recent meta-analysis, HPV is responsible for over 75% of penile intraepithelial neoplasia cases and over 50% of penile cancers [4]. Studies indicate that between 65% and 100% of sexually active individuals will be exposed to HPV at some point in their lives, regardless of the anatomical site (such as anal, oral, or genital) [5].

In addition to its association with anal and penile cancers, HPV has also been identified as a causative factor in various types of malignancies affecting the head and neck region. HPV infection is responsible for a minimum of 3% of oral cancer cases, 12% of pharyngeal cancer cases, and a significant proportion ranging, from 30% to 60%, of oropharyngeal carcinoma cases [6]. Men who have sex with men (MSM) represent a vulnerable population with an elevated risk of acquiring HPV infection compared to the general population [7,8]. Furthermore, previous studies reported that MSM have a higher prevalence of HPV-related cancer resulting from HPV infection when compared to women and heterosexual men [9,10].

In Malaysia, cervical cancer ranks as the fourth most prevalent cancer [11] and stands as the third leading cause of cancer-related mortality among women [12]. Furthermore, in addition to a high prevalence of HPV among women, anogenital HPV infection is also notably prevalent in men. A study conducted in Malaysia revealed that the overall prevalence of anogenital HPV infection, encompassing 14 high-risk genotypes and 2 low-risk wart-associated genotypes, was alarmingly high, reaching 29.6% among the healthy heterosexual male population [13]. Notably, this prevalence is highest among men in the young age group (18–24 years), where approximately one-third of individuals are affected [13], highlighting the particularly concerning rates of HPV infection in this demographic.

The implementation of HPV vaccination programs has demonstrated effectiveness in reducing the prevalence of HPV-associated diseases, thereby reducing the disease burden. Various factors may influence the acceptance of the HPV vaccine among MSM. These factors include knowledge regarding HPV and the vaccine, awareness of the associated health risks, concerns regarding vaccine safety and efficacy, social and cultural influences, and access to healthcare services [14,15,16,17]. In a meta-analysis examining the acceptability of HPV vaccines among MSM, an overall moderate level of acceptance was observed [18]. However, few studies have specifically examined the attitudes and acceptance of the HPV vaccine among MSM, particularly in the Malaysian context. Malaysia is a multiethnic country in Southeast Asia with a Muslim majority. Previous research on the acceptability of the HPV vaccine in Malaysia has primarily focused on heterosexual populations or women [19,20]. This is unsurprising because, in a predominantly Muslim country in Southeast Asia, marginalization and concealment of MSM, especially Malay-Muslim men, are common [21]. However, studies on HPV vaccination among MSM should be prioritized given its relevance within the context of STIs [22,23]. Understanding the attitudes, beliefs, and barriers towards HPV vaccination within this population is crucial for designing targeted interventions and increasing vaccine uptake.

In 2010, Malaysia launched a national vaccination program with the objective of providing three doses of HPV vaccines to all 13-year-old girls. In 2015, the program transitioned to a two-dose regimen. Importantly, this program has achieved a population coverage rate of over 80% [24]. The main focus of the HPV vaccination in Malaysia is on schoolgirls. Within this vaccination program, girls aged 13 to 17 are eligible to receive the HPV vaccines at no cost. However, for men interested in getting the HPV vaccine, it may be necessary to procure it independently at their own expense.

The primary objective of this study is to evaluate HPV vaccination acceptance among MSM in Malaysia. In addition, the study will investigate knowledge and attitudes towards HPV and HPV vaccination acceptance among MSM. Our study hypothesizes that demographics, sexual behavior, sexual identity, and stigma in healthcare settings concerning MSM receiving the HPV vaccine may influence acceptance. The study seeks to inform the development of effective strategies to promote vaccine uptake among the MSM population.

## 2. Materials and Methods

### 2.1. Study Participants and Survey Design

A cross-sectional online survey was conducted from May 2019 to September 2022. Eligibility criteria for participants included: (i) being 18 years of age or older, (ii) being Malaysian citizens, and (iii) identifying as biologically male and reporting engaging in sexual activity with other men within the past 12 months. Several recruitment methods were utilized to engage participants, focusing on leveraging popular social media platforms within the MSM community in Malaysia, including platforms such as Grindr and Hornet. The survey link was strategically posted on these platforms, ensuring maximum visibility among the target audience. Furthermore, social media profiles of LGBT-friendly non-governmental organizations were utilized, and peer referrals were also employed to reach and engage potential participants. In addition, a network of peer referrals was established, encouraging participants to refer their peers to participate in the survey. These collective approaches were employed to ensure a diverse sample of MSM in Malaysia was reached and engaged in the study.

Interested individuals were given a web link to access the online survey hosted by Qualtrics (Provo, UT, USA) [25]. Before proceeding to start the survey, the online survey form displayed the inclusion criteria to ensure that only individuals who met the criteria were included in the study. Participants were also required to complete an Institutional Review Board (IRB)-approved online informed consent form before proceeding with the survey. On average, the survey took approximately 12 min to complete. Participation in the survey was voluntary, and participants did not receive monetary compensation.

### 2.2. Sample Size Determination

The sample size was calculated using the formula: *n* = Z^2^ × P × (1 − P)/d^2^ [26]. The sample size was calculated based on the following assumptions: a conservative vaccination intention of 50.0%, a margin of error of 0.05 (5%), and a 95% confidence level. The calculated sample size was 384 (n = [1.96^2^ × 0.5 × (1 − 0.5)]/0.05^2^).

### 2.3. Survey Instruments

A self-administered questionnaire in English was developed with reference to the relevant previous studies in the literature [27,28,29]. The questionnaire underwent content validation by a panel of experts consisting of both academicians and researchers who possess prior experience in studies related to HPV vaccine hesitancy. The instrument underwent a pilot test with 20 individuals. No revisions were made to the questionnaire or the implementation procedures following the pilot test. The survey consisted of questions that assessed (1) demographic background, sexual identity, sexual behaviors, and symptoms and history of STIs; (2) knowledge related to HPV and HPV infection; (3) attitudes towards HPV infection and HPV vaccination; (4) stigma in healthcare settings surrounding the vaccination against HPV for gay and bisexual men, and (5) HPV vaccination intention.

Participant demographic characteristics collected in the study included age, ethnicity, highest educational attainment, occupation, and income. Participants self-reported their sexual identity, sexual behaviors, symptoms of STIs, and any history of being diagnosed with an STI.

To evaluate the participants’ knowledge regarding HPV and HPV infections, a 13-item questionnaire was utilized in this study. The questionnaire required participants to indicate whether a given statement was “true”, “false”, or “don’t know”. For scoring purposes, a correct response was assigned a value of one, while an incorrect or “don’t know” response received a value of zero. The scores for each item were summed up, and those who scored equal to and higher than the median value were classified as having good knowledge, while those who scored lower were classified as having poor knowledge.

To assess the attitude of the participants towards HPV infection, a six-item questionnaire was employed in this study. The questionnaire in this study utilized a 4-point Likert scale to measure participant responses. The scale included options ranging from “very high” to “very low” and from “strongly disagree” to “strongly agree”. Attitudes towards HPV vaccination were measured using 11-item questions. Similarly, a 4-point Likert scale was used to measure participant responses. The scale included options ranging from “strongly disagree” to “strongly agree”.

Participants were queried regarding the perception of stigma and discrimination in healthcare settings surrounding the vaccination against HPV for gay and bisexual men, with the options for response being either “yes” or “no”. The acceptance of the HPV vaccine was assessed using a rating scale ranging from 1 to 7, with 1 indicating “no chance” of receiving the vaccine and 7 indicating a “certainty to happen”. Participants were asked to indicate their intention to receive the HPV vaccine based on this scale. The study questionnaire is provided in Supplementary File S1.

### 2.4. Ethical Considerations

This study was approved by the University of Malaya Research Ethics Committee (UMREC). Approval code: (UMREC)-UM.TNC2/UMREC—493.

### 2.5. Statistical Analysis

Frequency tables, charts, and proportions were used for data summarization. The proportion and its respective 95% confidence interval (CI) were calculated. Cronbach’s alpha was calculated for the knowledge items to assess reliability in terms of internal consistency. The knowledge scale had good internal consistency, with a Cronbach’s alpha of 0.919. Univariate analyses followed by a multivariable logistic regression analysis, including all factors showing significance (*p* < 0.05), were conducted to determine factors associated with the intention to take the HPV vaccine. Odds ratios (OR), 95% confidence intervals (95% CI), and *p*-values were calculated for each independent variable. The “certain to happen”, “very likely”, and “likely” categories were merged to denote the intent category. The “no chance”, “very unlikely”, “unlikely”, and “moderate chance” categories were merged to denote the non-intent reference category. A Hosmer–Lemeshow test was performed to check the model’s goodness of fit [30]. All statistical analyses were performed using the Statistical Package for the Social Sciences version 20.0 (IBM Corp., Armonk, NY, USA). A *p*-value of less than 0.05 was considered statistically significant.

## 3. Results

A total of 503 responses were received and 62 participants reported prior receipt of the HPV vaccine and were excluded from the analysis. The final sample for analysis yielded 441 participants. Of this sample, 34.2% belonged to the age group of 24 to 29 years, while 26.3% fell within the age range of 30 to 35 years. The majority of participants were of Chinese ethnicity, accounting for 48.5% of the sample, followed by Malay participants, comprising 42.0% of the total. A significant 74.6%, had received tertiary education, while 16.6% held postgraduate degrees. In terms of occupation, more than half of the participants (53.5%) belonged to the professional and managerial group (Table 1).

Most participants (73.7%) self identified as gay, homosexual, or belonging to people like us (PLU) as their sexual orientation. Under half of the participants (48.3%) reported engaging in condomless anal intercourse within the past six months. Additionally, 25.2% of the participants reported experiencing symptoms of STIs at some point, while 26.8% had a history of STIs within the past year.

**Figure 1:**
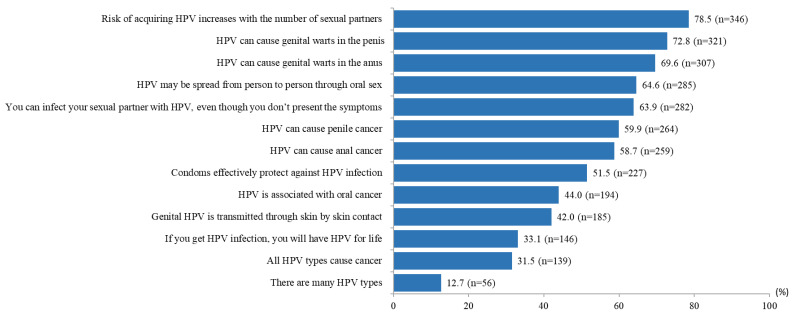
Proportion of correct responses for knowledge items (N = 441).

Figure 1 shows the proportion of correct responses for knowledge items. The majority of participants (78.5%) accurately responded that having more sexual partners increases the risk of developing HPV. A considerable proportion, 72.8% and 69.6%, respectively, were aware that HPV infections can lead to genital warts in the penis and anus. A total of 63.9% of study participants were aware that HPV infection can occur without presenting any symptoms. However, 48.5% mistakenly believed that condoms fully protect against HPV infection although HPV can be transmitted via skin-to-skin contact. A high proportion of participants lacked knowledge regarding the lifelong nature of HPV infection (66.9%). The average knowledge score was 6.82 (SD = 3.93, range 0–13). The median was 8.0 (interquartile range [IQR], 4 to 10). The knowledge scores were categorized as a score of 8–13 or 0–7, based on the median split; as such, a total of 232 (52.6%; 95% CI 47.8 to 57.4) were categorized as having a score of 8–13 and 209 (47.4%; 95% CI 42.6 to 52.2) as having a score of 0–7.

As indicated in Table 1, most participants disagreed that HPV infection risk is low (71.0%). However, a considerable proportion (64.2%) perceived low/very low risk of getting HPV infection; 96.8% expressed disagreement that diseases from HPV infections are not serious (96.8%) and that HPV infection poses a greater problem for women than it does for men (92.3%).

With regard to attitudes concerning HPV vaccination, 69.2% reported that they lacked sufficient information about the HPV vaccine to make a decision regarding vaccination. A majority of participants also concurred that the long-term potential side effects of HPV vaccination remain unclear. Approximately 32.7% perceived taking HPV vaccination as a sign of promiscuity, while 47.6% acknowledged that HPV vaccination is a sensitive topic. A considerable number of participants expressed agreement that recommendations for HPV vaccines from friends (70.8%) and healthcare providers (69.2%) can positively influence their intention to get vaccinated against HPV. The majority of participants (75.4%) anticipate the presence of stigma in healthcare settings when it comes to gay and bisexual men receiving the HPV vaccine.

**Figure 2:**
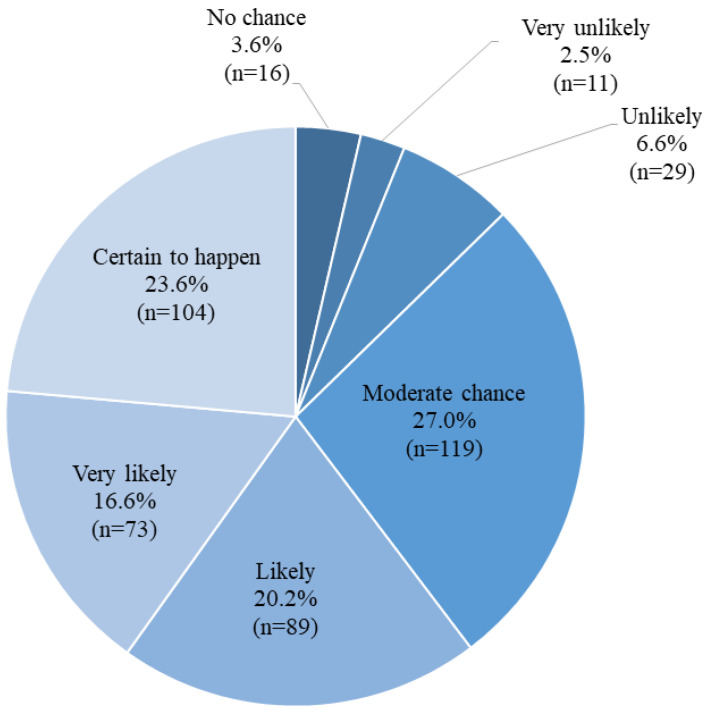
Proportion of responses for intention to take HPV vaccine (N = 441).

Figure 2 presents the responses regarding the intention to receive the HPV vaccine. A total of 27.0% indicated a “moderate chance”, 6.6% expressed “unlikely”, and 2.5% reported feeling “very unlikely” to receive the HPV vaccine. In total, there were 266 (60.4%) responses in the intent category (responded “certain to happen”, “very likely”, and “likely”).

Supplementary Table S1 shows the univariate analyses of factors influencing the intention to receive the HPV vaccine in two subgroups. Univariate analyses that showed significant associations were included in the multivariable logistic regression. The multivariable logistic regression analysis showed no significant association between demographic variables and HPV vaccination intention (Table 1). However, sexual identity was found to be a strong predictor of HPV vaccine acceptance. Participants who identify themselves as bisexual (OR 6.93, 95% CI 2.35–20.41) and gay/homosexual (OR 4.36, 95% CI 1.66–11.42) reported higher intention to receive the HPV vaccine than those who are heterosexual. The multivariate logistic model also showed that having the intention of HPV vaccination was positively and significantly associated with a high level of knowledge (OR 1.79, 95% CI 1.09–2.95). None of the attitudes towards HPV infection and HPV vaccination items were significantly associated with HPV vaccination intention in the multivariate logistic regression model. Although a relatively high proportion (75.7%) of individuals anticipated stigma in healthcare settings regarding HPV vaccination for gay and bisexual men, it was not found to be significantly associated with the intention to be vaccinated for HPV infection in the multivariate logistic regression model.

## 4. Discussion

The MSM population faces a high risk of HPV infection, and targeted HPV vaccination is the most cost-effective public health measure for prevention. Studying HPV vaccine acceptance among the MSM population in Malaysia, a country with a Muslim majority and conservative values, is crucial because it sheds light on the potential challenges and opportunities associated with implementing effective HPV prevention strategies in such cultural and social contexts.

The knowledge score from this study reveals gaps in participants’ understanding of the transmission of HPV. For example, a significant number of participants were unaware that genital HPV can be transmitted through skin contact or non-sexual contact [31]. Many were unaware that HPV infection can be asymptomatic and lifelong. Additionally, even when a condom is used, certain HPV strains can induce genital warts or cervical neoplasia. Although condoms provide some level of protection, they do not eliminate the risk [32]. Regular HPV vaccination and practicing safer sexual behaviors can further reduce the risk of HPV-related complications. Hence, targeted educational interventions are required to address these knowledge gaps and increase awareness of HPV infections and their consequences.

In this study, participants perceived low susceptibility to HPV infection, even though they recognized its severity. The findings regarding attitudes toward HPV infection underscore the importance of emphasizing the risks associated with HPV infections. Low-risk perception and susceptibility have been identified as barriers to HPV vaccine acceptance among men and MSM [33,34,35]. While the study did not find a significant association between attitudes and intention for HPV vaccination, the low-risk perception of HPV infections found in the study requires attention and should be addressed through targeted intervention and awareness campaigns.

In general, the participants showed varied attitudes to HPV vaccination. This study underscores the heightened risk of HPV infection among MSM. It is evident that a substantial proportion of MSM expressed a moderate willingness to receive the HPV vaccine, underscoring the need for intensified efforts to promote and encourage HPV vaccination within this demographic. The research underscores the pivotal role of knowledge in influencing vaccination willingness among MSM. While attitudes toward HPV infection and vaccination may not be significant predictors of vaccination intention, certain misconceptions have been identified. Therefore, to enhance vaccination rates, it is crucial to prioritize the dissemination of accurate information about HPV and the HPV vaccine while fostering positive attitudes.

Many participants reported that inadequate information about the HPV vaccine hindered their decision-making in vaccination. Additionally, the long-term potential side effects of HPV vaccination remain unclear for many participants. HPV vaccination has been available for a significant period, but study participants highlighted that the long-term potential side effects of HPV vaccination remain unclear. Therefore, awareness interventions that provide accurate information about the safety of HPV vaccination with supporting clinical data are crucial in promoting public understanding and acceptance of the vaccine. Most importantly, intervention should enlighten participants that HPV vaccines have undergone extensive clinical testing and have been proven safe and effective in preventing HPV infections. Numerous large-scale clinical trials involving thousands of participants have been conducted to evaluate the safety of HPV vaccines before they were approved for public use. Additionally, the HPV vaccine’s safety has been confirmed through post-marketing surveillance studies and by data from national immunization programs [36]. Of important note, research findings have indicated that the HPV vaccine may potentially expedite the clearance of existing HPV infections in women [37]. The early clearance of pre-existing infections holds significant advantages, as it not only diminishes the risk of HPV-related diseases but also disrupts the transmission cycle within populations. This effect can wield a profound influence on public health by further diminishing the prevalence of HPV infections. Further research is warranted to comprehensively assess the potential positive impact of HPV vaccination on men who are already HPV-positive in terms of infection clearance, thereby providing empirical evidence of the supplementary benefits conferred by HPV vaccination in individuals who have previously contracted the virus. Collaboration with healthcare providers, community leaders, and educators to disseminate this information can further enhance the effectiveness of awareness interventions. Our findings also suggest that fostering positive recommendations for HPV vaccines from men who have received HPV vaccination and healthcare providers can be a powerful strategy for increasing vaccination rates.

Addressing sensitivity towards HPV vaccination is crucial in Muslim countries, where sexual-related issues (especially homosexuality) remain a cultural taboo [23]. In this study, many participants anticipated stigma in healthcare settings regarding HPV vaccination for MSM. In addition, it was found that a small minority, comprising approximately one-third of the participants, expressed the belief that taking the HPV vaccination is indicative of promiscuity and considered that HPV vaccination is a sensitive topic. These findings underscore the importance of implementing comprehensive strategies aimed at addressing sensitivity issues and enhancing the acceptance of HPV vaccination. A culturally sensitive approach is essential in dispelling misconceptions about the vaccine’s association with promiscuity. Integrating HPV vaccination with MSM-related services such as STI counseling and testing can improve accessibility and acceptability among this population. Ultimately, the results of this study carry significant implications for public health interventions targeting HPV prevention and control strategies among MSM in Malaysia. It is crucial to acknowledge that attitudes and motivations for HPV immunization among MSM can vary considerably among diverse cultural populations, with social and cultural factors exerting a significant influence on individuals’ perceptions of HPV vaccination. Notably, in certain Western nations where homosexuality is more culturally accepted, HPV vaccination acceptance tends to be higher [38,39]. While it is undeniable that populations with diverse cultural backgrounds pose challenges to MSM HPV vaccine acceptance, some countries with progressive attitudes towards MSM identities have taken proactive steps by implementing tailored HPV vaccination programs to address the heightened risk of HPV-related diseases [38,40]. However, it remains a challenge for Muslim-majority countries to implement such programs for MSM due to cultural and societal factors. In light of these challenges, one possible suggestion would be to engage in culturally sensitive dialogue and collaboration with local religious and community leaders to promote awareness and acceptance of HPV vaccination, emphasizing its broader benefits in preventing cancer. Additionally, providing education and information campaigns that respect cultural norms and values while highlighting the importance of vaccination for health could help bridge the gap in HPV vaccine acceptance among MSM in these regions.

The study participants displayed a moderate level of acceptability towards the HPV vaccine, with approximately 60% of them falling into the category of intending to vaccinate. Our study adds to the evidence indicating that MSM have an average acceptance of HPV vaccination [18]. No significant differences in HPV vaccine acceptance were observed based on demographic backgrounds. However, bisexual individuals expressed a higher intention for HPV vaccination compared to homosexual/gay individuals. The finding that bisexual individuals exhibited a higher intention for HPV vaccination than homosexual/gay individuals suggests that sexual orientation may play a role in shaping attitudes and decisions regarding HPV vaccination. It is possible that bisexual individuals, due to their diverse sexual orientation, may perceive themselves to be at higher risk of contracting HPV and, therefore, place greater importance on preventive measures like vaccination. This finding emphasizes the importance of tailoring public health interventions to consider the unique perspectives and needs of various sexual orientations.

In this study, a higher level of knowledge significantly predicted HPV vaccine acceptance. Similarly, numerous other studies have consistently reported that an increased awareness of HPV is positively associated with a greater willingness to undergo vaccination among men [33]. The findings of this study, along with evidence from other studies, highlight the importance of increasing knowledge and awareness regarding HPV vaccination among MSM. Based on this finding, we recommend the implementation of targeted educational programs to increase knowledge about HPV and its vaccination. The information provided by these programs should be accurate and exhaustive, as empowering MSM with knowledge about HPV and its vaccination plays a crucial role in improving overall HPV vaccination rates.

Several study limitations should be acknowledged. Firstly, the use of an online survey may introduce bias, as participation relies on self-selection and access to the internet. Consequently, individuals who lack internet access or do not engage in social media may be underrepresented in the sample. Secondly, the study employed convenience sampling, further potentially biasing the sample toward individuals who are more active online or open about their sexual orientation. Moreover, the data collected may be influenced by reporting bias, as participants may be hesitant to divulge sensitive information related to their sexual behaviors and attitudes. These limitations, coupled with the relatively small sample size of 441 participants, pose challenges in generalizing the findings to the broader MSM population in Malaysia, especially given the unknown size of this population. Despite these limitations, this study provides valuable insights that can inform future research and interventions aimed at enhancing HPV vaccination rates among the MSM population in Malaysia.

## 5. Conclusions

MSM are at an elevated risk of HPV infection. The study indicates that most MSM showed a moderate willingness to be vaccinated against HPV, indicating the need to enhance efforts to promote and encourage HPV vaccination among this population. In this study, knowledge played a crucial role in influencing vaccination willingness among MSM. While attitudes towards HPV infection and vaccination may not be significant predictors of vaccination intention, some misconceptions were identified in the study. Therefore, to increase vaccination rates, it is important to prioritize the promotion of HPV and HPV vaccine knowledge, alongside enhancing positive attitudes. The findings emphasize the need for comprehensive strategies to address sensitivity issues and enhance the acceptance of HPV vaccination. A culturally sensitive approach is essential in dispelling misconceptions about the vaccine’s association with promiscuity. HPV vaccination can be combined with MSM-related services, such as STI counseling and testing, to enhance accessibility and acceptability among this population. The study’s findings carry significant implications for public health interventions targeting HPV prevention and control strategies for MSM in Malaysia.

## Figures and Tables

**Table 1:** Factors associated with intention to receive HPV vaccine (N = 441).

		Multivariable Analysis
		Intention to Receive HPV Vaccine in the Next Year
	N (%)	Intent vs. Non-Intent ^¶^
		aOR (95% CI)
Socio-demographic characteristics		
Ethnicity		
Malay	185 (42.0)	1.73 (0.62–4.81)
Chinese	214 (48.5)	0.69 (0.25–1.87)
Indian	15 (3.4)	1.15 (0.25–5.34)
Bumiputera Sabah/Sarawak/Others	27 (6.1)	Reference
Sexual characteristics		
Sexual identity		
Bisexual	82 (18.6)	6.93 (2.35–20.41) ***
Gay/Homosexual/PLU	325 (73.7)	4.36 (1.66–11.42) **
Heterosexual/straight	27 (6.1)	Reference
Queer/gender queer †	7 (1.6)	-
Symptoms related to sexually transmitted infections in the last year		
Yes	111 (25.2)	1.04 (0.45–2.42)
No	330 (74.8)	Reference
History of sexually transmitted infections in the last year		
Yes	118 (26.8)	1.10 (0.50–2.43)
No	323 (73.2)	Reference
Knowledge related to HPV and HPV infection		
Total knowledge score		
Low score (0–7)	209 (47.4)	Reference
High score (8–13)	232 (52.6)	1.79 (1.09–2.95) *
Attitudes towards HPV infection		
Level of chance of getting HPV infection		
Very high/High	158 (35.8)	1.71 (0.92–3.18)
Low/Very low	283 (64.2)	Reference
Level of worriedness about getting the disease from HPV infection		
Very high/High	250 (56.7)	1.63 (0.93–2.85)
Low/Very low	191 (43.3)	Reference
The chance of HPV infection seems small to me		
Strongly agree/Agree	128 (29.0)	Reference
Disagree/Strongly disagree	313 (71.0)	1.32 (0.74–2.36)
The diseases I may get due to HPV infections are not serious		
Strongly agree/Agree	14 (3.2)	Reference
Disagree/Strongly disagree	427 (96.8)	3.32 (0.85–12.94)
Attitudes towards HPV vaccination		
I do not have enough information about HPV vaccine to decide on vaccination		
Strongly agree/Agree	305 (69.2)	0.89 (0.43–1.84)
Disagree/Strongly disagree	136 (30.8)	Reference
The long-term potential side effects of HPV vaccination remain unclear		
Strongly agree/Agree	312 (70.7)	1.16 (0.55–2.45)
Disagree/Strongly disagree	129 (29.3)	Reference
I find HPV vaccination a sensitive topic because it has to do with sexual activity		
Strongly agree/Agree	210 (47.6)	0.75 (0.45–1.25)
Disagree/Strongly disagree	231 (52.4)	Reference
I am confident that I could take up HPV vaccines if I want to		
Strongly agree/Agree	349 (79.1)	2.56 (0.88–7.50)
Disagree/Strongly disagree	92 (20.9)	Reference
I will take HPV vaccine if doctor recommends me to take up HPV vaccines		
Strongly agree/Agree	364 (82.5)	1.78 (0.42–7.49)
Disagree/Strongly disagree	77 (17.5)	Reference
I will take HPV vaccine if my friends recommend me to take up HPV vaccines		
Strongly agree/Agree	329 (74.6)	1.99 (0.89–4.41)
Disagree/Strongly disagree	112 (25.4)	Reference

† Exluded from multivariable analysis due to the number of samples less than 10; * *p* < 0.05, ** *p* < 0.01, *** *p* < 0.001; Hosmer–Lemeshow test, chi-square: 12.145, *p*-value: 0.145; Nagelkerke R^2^: 0.373; ^¶^ Intent = certain to happen, very likely, likely; Non-intent = no chance, very unlikely, unlikely, moderate chance.

## Data Availability

The data presented in this study are available upon request from the corresponding author.

## Supplementary Materials

The following supporting information can be downloaded at: https://www.mdpi.com/article/10.3390/pathogens12101261/s1, Supplementary File S1. Survey Questionnaire. Supplementary Table S1. Univariable analysis of factors associated with intention to receive HPV vaccine in the next year (N = 441).
