# Supplementary material for: Factors Influencing HPV Vaccine Intentions in Malaysian Men Who Have Sex with Men: A Cross-Sectional Study in Malaysia
Source: Pathogens. 2023 Oct 19;12(10):1261. doi: 10.3390/pathogens12101261 (PMC10610168; doi:10.3390/pathogens12101261)
Supplement: Supplementary file 1 [file pathogens-12-01261-s001.zip › pathogens-2637650-supplementary.pdf]

Supplementary File S1. Survey Questionnaire.

ACCEPTABILITY OF THE HUMAN PAPILLOMAVIRUS (HPV) VACCINE AMONG MEN WHO HAVE SEX WITH MEN (MSM) IN MALAYSIA

SECTION A: GENERAL INFORMATION

1	Age	______________________ years old
2	Ethnicity	[1] Malay<br>[2] Chinese<br>[3] Indian<br>[4] Bumiputera Sabah/Sarawak<br>[5] Others, please specify__________________
3	Occupation	[1] Professional and managerial<br>[2] Skilled worker<br>[3] Non-skilled worker<br>[4] Retired<br>[5] Unemployed<br>[6] Others, please specify__________________
4	Highest educational attainment	[1] No formal education<br>[2] Primary school<br>[3] Secondary school<br>[4] College/Bachelor<br>[5] Postgraduate
5	Average monthly income	[1] MYR1,000 and below<br>[2] 1001–2000<br>[3] 2001–3000<br>[4] 3001–4000<br>[5] 4001–5000<br>[6] 5001–6000<br>[7] 6001–7000<br>[8] 7001–8000<br>[9] 8001–9000<br>[10] 9001–10,000<br>[11] More than MYR10,000

SECTION B: SEXUAL IDENTITY AND BEHAVIOR

1	Sexual identity?	[1] Bisexual<br>[2] Gay/homosexual/PLU<br>[3] Heterosexual/straight<br>[4] Queer/gender queer
2	Anal intercourse without condoms with male sexual partner(s) in the last six months.	[1] Yes<br>[2] No
3	Symptoms related to sexually transmitted infections (STIs) in the last one year	[1] Yes<br>[2] No
4	History of sexually transmitted infections in the last one year	[1] Yes<br>[2] No

SECTION C: KNOWLEDGE RELATED TO HPV AND HPV VACCINATION

The following are questions about your views on Human Papilloma Virus (HPV), please answer "True", "False", or "Don't know" to each statement

1	HPV can cause genital warts in the penis	[1] True [2] False [3] Don't know
2	HPV can cause genital warts in the anus	[1] True [2] False [3] Don't know
3	HPV can cause penile cancer	[1] True [2] False [3] Don't know
4	HPV can cause anal cancer	[1] True [2] False [3] Don't know
5	HPV is associated with oral cancer	[1] True [2] False [3] Don't know
6	HPV may be spread from person to person through oral sex	[1] True [2] False [3] Don't know
7	Genital HPV is transmitted through skin-to-skin contact	[1] True [2] False [3] Don't know
8	You can infect your sexual partner with HPV, even	[1] True [2] False [3] Don't know

	though you don’t present the symptoms	
9	Risk of acquiring HPV increases with the number of sexual partners	[1] True [2] False [3] Don’t know
10	If you get HPV infection, you will have HPV for life	[1] True [2] False [3] Don’t know
11	Condoms protect against HPV infection	[1] True [2] False [3] Don’t know
12	There are many HPV types	[1] True [2] False [3] Don’t know
13	All HPV types cause cancer	[1] True [2] False [3] Don’t know

SECTION D: ATTITUDES TOWARD HPV INFECTION AND HPV VACCINATION

Attitudes towards HPV infection					
1	Please rate your chance of getting HPV infection	[1] Very low	[2] Low	[3] High	[4] Very high
2	What is your level of worriedness about the diseases that you will get from HPV infection?	[1] Very low	[2] Low	[3] High	[4] Very high
	Please rate the level of agreement to the following statements				
3	The chance of HPV infection seems small to me	[1] Totally disagree	[2] Disagree	[3] Agree	[4] Totally agree
4	HPV infection is not important enough to get vaccinated against	[1] Totally disagree	[2] Disagree	[3] Agree	[4] Totally agree
5	The diseases I may get due to HPV infection are not serious	[1] Totally disagree	[2] Disagree	[3] Agree	[4] Totally agree
6	HPV infection is a bigger problem for women than it is for men	[1] Totally disagree	[2] Disagree	[3] Agree	[4] Totally agree
Attitudes toward HPV vaccination					
7	Getting vaccinated against HPV has more drawbacks than benefits	[1] Totally disagree	[2] Disagree	[3] Agree	[4] Totally agree
8	I am against vaccination in general	[1] Totally disagree	[2] Disagree	[3] Agree	[4] Totally agree
9	I don't have enough information about HPV vaccine to	[1] Totally disagree	[2] Disagree	[3] Agree	[4] Totally agree

	decide on vaccination				
10	The possible side effects of HPV vaccination in the long term are not clear to me	[1] Totally disagree	[2] Disagree	[3] Agree	[4] Totally agree
11	It is embarrassing for men to take up HPV vaccines	[1] Totally disagree	[2] Disagree	[3] Agree	[4] Totally agree
12	It is troublesome to take up HPV vaccines	[1] Totally disagree	[2] Disagree	[3] Agree	[4] Totally agree
13	Taking up an HPV vaccine may be a sign of promiscuity	[1] Totally disagree	[2] Disagree	[3] Agree	[4] Totally agree
14	I find HPV vaccination a sensitive topic because it has to do with sexual activity	[1] Totally disagree	[2] Disagree	[3] Agree	[4] Totally agree
15	I am confident that I could take up HPV vaccines if I wanted to	[1] Totally disagree	[2] Disagree	[3] Agree	[4] Totally agree
16	I will take an HPV vaccine if a doctor recommends me to take up HPV vaccines	[1] Totally disagree	[2] Disagree	[3] Agree	[4] Totally agree
17	I will take an HPV vaccine if my friends recommends me to take up HPV vaccines	[1] Totally disagree	[2] Disagree	[3] Agree	[4] Totally agree

SECTION E: STIGMA

1	Do you anticipate stigma in health care settings for gay and bisexual men who receive the HPV vaccine?	[1] Yes<br>[2] No

SECTION F: VACCINATION INTENTION

Currently in Malaysia, the vaccine for HPV is available for boys and men and it has been shown to prevent genital warts and may help prevent certain HPV-related cancers. The vaccine requires 3 doses given over 6 months. Please rate your intention to get an HPV vaccination?

1	Please indicate your intention to receive the HPV vaccine on a scale of 1 to 7, with 1 being no chance and 7 being certain to happen.	[7] Certain to happen<br>[6] Very likely<br>[5] Likely<br>[4] Moderate chance<br>[3] Unlikely<br>[2] Very unlikely<br>[1] No chance

Appendix 2 Univariable analysis of factors associated with intention to receive HPV vaccine in the next year (N=441)

		Univariable analysis		
		Intention to receive HPV vaccine		
	N (%)	Intent (n=266)	Non-intent (n=175)	p-value
Socio-demographic characteristics				
Age group (years)				
18–23	106 (24.0)	58 (21.8)	48 (27.8)	0.482
24–29	151 (34.2)	97 (36.5)	54 (30.9)	
30–35	116 (26.3)	71 (26.7)	45 (25.7)	
>35	68 (15.4)	40 (15.0)	28 (16.0)	
Ethnicity				
Malay	185 (42.0)	132 (49.6)	53 (30.3)	0.001
Chinese	214 (48.5)	109 (41.0)	105 (60.0)	
Indian	15 (3.4)	8 (3.0)	7 (4.0)	
Bumiputera	27 (6.1)	17 (6.4)	10 (5.7)	
Sabah/Sarawak/Others				

Highest educational attainment				
Secondary school and below	39 (8.8)	25 (9.4)	14 (8.0)	0.834
Diploma/Bachelor's degree	329 (74.6)	196 (73.7)	133 (76.0)	
Postgraduate degree	73 (16.6)	45 (16.9)	28 (16.0)	
Occupation				
Professional and managerial	236 (53.5)	143 (53.8)	93 (53.1)	0.072
Skilled worker	48 (10.9)	31 (11.7)	17 (9.7)	
Non-skilled worker/ Self-employed	32 (7.3)	24 (9.0)	8 (4.6)	
Student	55 (12.5)	35 (13.2)	20 (11.4)	
Retired/Unemployed	70 (15.9)	33 (12.4)	37 (21.1)	
Monthly income (MYR)				
1000 and below	112 (25.4)	64 (24.1)	48 (27.4)	0.429
1001-4000	167 (37.9)	109 (41.0)	58 (33.1)	
4001-8000	111 (25.2)	64 (24.1)	47 (26.9)	
80001 and above	51 (11.6)	29 (10.9)	22 (12.6)	
Sexual characteristics				
Sexual identity				
Bisexual	82 (18.6)	58 (21.8)	24 (13.7)	0.006
Gay/Homosexual/PLU	325 (73.7)	194 (72.9)	131 (74.9)	
Heterosexual/straight	27 (6.1)	9 (3.4)	18 (10.3)	
Queer/gender queer †	7 (1.6)	5 (1.9)	2 (1.1)	
Sexual behaviour				
Engaging in anal intercourse without condom with male sexual partner in the past 6 months				
Yes	213 (48.3)	135 (50.8)	78 (44.6)	0.207
No	228 (51.7)	131 (49.2)	97 (55.4)	
Symptoms related to sexually transmitted infections in the last year				
Yes	111 (25.2)	82 (30.8)	29 (16.6)	0.001
No	330 (74.8)	184 (69.2)	146 (83.4)	
History of sexually transmitted infections in the last year				
Yes	118 (26.8)	86 (32.3)	32 (18.3)	0.001
No	323 (73.2)	180 (67.7)	143 (81.7)	
Knowledge related to HPV and HPV infection				
Total knowledge score				
Low score (0–7)	209 (47.4)	97 (36.5)	112 (64.0)	p<0.0001
High score (8–13)	232 (52.6)	169 (63.5)	63 (36.0)	

Attitudes towards HPV infection				
Level of chance of getting HPV infection				
Very high/High	158 (35.8)	126 (47.4)	32 (18.3)	p<0.0001
Low/Very low	283 (64.2)	140 (52.6)	143 (81.7)	
Level of worriedness about getting disease from HPV infection				
Very high/High	250 (56.7)	189 (71.1)	61 (34.9)	p<0.0001
Low/Very low	191 (43.3)	77 (28.9)	114 (65.1)	
The chance of HPV infection seems small to me				
Strongly agree/Agree	128 (29.0)	67 (25.2)	61 (34.9)	0.032
Disagree/Strongly disagree	313 (71.0)	199 (74.8)	114 (65.1)	
HPV infection is not important enough to get vaccinated against				
Strongly agree/Agree	46 (10.4)	23 (8.6)	23 (13.1)	0.152
Disagree/Strongly disagree	395 (89.6)	243 (91.4)	152 (86.9)	
The diseases I may get due to HPV infections are not serious				
Strongly agree/Agree	14 (3.2)	4 (1.5)	10 (5.7)	0.023
Disagree/Strongly disagree	427 (96.8)	262 (98.5)	165 (94.3)	
HPV infection is a bigger problem for women than it is for men				
Strongly agree/Agree	34 (7.7)	17 (6.4)	17 (9.7)	0.207
Disagree/Strongly disagree	407 (92.3)	249 (93.6)	158 (90.3)	
Attitudes towards HPV vaccination				
Getting vaccinated against HPV has more drawbacks than benefits				
Strongly agree/Agree	22 (5.0)	10 (3.8)	12 (6.9)	0.180
Disagree/Strongly disagree	419 (95.0)	256 (96.2)	163 (93.1)	
I am against vaccination in general				
Strongly agree/Agree	15 (3.4)	7 (2.6)	8 (4.6)	0.293
Disagree/Strongly disagree	426 (96.6)	259 (97.4)	167 (95.4)	
I do not have enough information about HPV vaccine to decide on vaccination				
Strongly agree/Agree	305 (69.2)	204 (76.7)	101 (57.7)	p<0.0001

Disagree/Strongly disagree	136 (30.8)	62 (23.3)	74 (42.3)	
The long-term potential side effects of HPV vaccination remain unclear				
Strongly agree/Agree	312 (70.7)	210 (78.9)	102 (58.3)	p<0.0001
Disagree/Strongly disagree	129 (29.3)	56 (21.1)	73 (41.7)	
It is embarrassing for men to take up HPV vaccines				
Strongly agree/Agree	120 (27.2)	70 (26.3)	50 (28.6)	0.662
Disagree/Strongly disagree	321 (72.8)	196 (73.7)	125 (71.4)	
It is troublesome to take up HPV vaccines				
Strongly agree/Agree	152 (34.5)	98 (36.8)	54 (30.9)	0.219
Disagree/Strongly disagree	289 (65.5)	168 (63.2)	121 (69.1)	
Taking up HPV vaccines may be a sign of promiscuity				
Strongly agree/Agree	144 (32.7)	96 (36.1)	48 (27.4)	0.062
Disagree/Strongly disagree	297 (67.3)	170 (63.9)	127 (72.6)	
I find HPV vaccination a sensitive topic because it has to do with sexual activity				
Strongly agree/Agree	210 (47.6)	143 (53.8)	67 (38.3)	0.002
Disagree/Strongly disagree	231 (52.4)	123 (46.2)	108 (61.7)	
I am confident that I could take up HPV vaccines if I wanted to				
Strongly agree/Agree	349 (79.1)	244 (91.7)	105 (60.0)	p<0.0001
Disagree/Strongly disagree	92 (20.9)	22 (8.3)	70 (40.0)	
I will take an HPV vaccine if a doctor recommends me to take up HPV vaccines				
Strongly agree/Agree	364 (82.5)	252 (94.7)	112 (64.0)	p<0.0001
Disagree/Strongly disagree	77 (17.5)	14 (5.3)	63 (36.0)	
I will take an HPV vaccine if my friends recommends me to take up HPV vaccines				
Strongly agree/Agree	329 (74.6)	233 (87.6)	96 (54.9)	p<0.0001
Disagree/Strongly disagree	112 (25.4)	33 (12.4)	79 (45.1)	
Stigma				
I anticipate stigma in healthcare settings surrounding HPV vaccination for gay and bisexual men				
Yes	334 (75.7)	199 (74.8)	135 (77.1)	0.650
No	107 (24.3)	67 (25.2)	40 (22.9)	

Supplementary Table S1. Univariable analysis of factors associated with intention to receive HPV vaccine in the next year (N=441).

		Univariable analysis		
		Intention to receive HPV vaccine in the next year		
	N (%)	Intent (n=266)	Non-intent (n=175)	p-value
Socio-demographic characteristics				
Age group (years)				
18–23	106 (24.0)	58 (21.8)	48 (27.8)	0.482
24–29	151 (34.2)	97 (36.5)	54 (30.9)	
30–35	116 (26.3)	71 (26.7)	45 (25.7)	
>35	68 (15.4)	40 (15.0)	28 (16.0)	
Ethnicity				
Malay	185 (42.0)	132 (49.6)	53 (30.3)	0.001
Chinese	214 (48.5)	109 (41.0)	105 (60.0)	
Indian	15 (3.4)	8 (3.0)	7 (4.0)	
Bumiputera Sabah/Sarawak/Others	27 (6.1)	17 (6.4)	10 (5.7)	
Highest educational attainment				
Secondary school and below	39 (8.8)	25 (9.4)	14 (8.0)	0.834
Diploma/Bachelor's degree	329 (74.6)	196 (73.7)	133 (76.0)	
Postgraduate degree	73 (16.6)	45 (16.9)	28 (16.0)	
Occupation				
Professional and managerial	236 (53.5)	143 (53.8)	93 (53.1)	0.072
Skilled worker	48 (10.9)	31 (11.7)	17 (9.7)	
Non-skilled worker/ Self-employed	32 (7.3)	24 (9.0)	8 (4.6)	
Student	55 (12.5)	35 (13.2)	20 (11.4)	
Retired/Unemployed	70 (15.9)	33 (12.4)	37 (21.1)	
Monthly income (MYR)				
1000 and below	112 (25.4)	64 (24.1)	48 (27.4)	0.429
1001–4000	167 (37.9)	109 (41.0)	58 (33.1)	
4001–8000	111 (25.2)	64 (24.1)	47 (26.9)	
80001 and above	51 (11.6)	29 (10.9)	22 (12.6)	
Sexual characteristics				
Sexual identity				
Bisexual	82 (18.6)	58 (21.8)	24 (13.7)	0.006
Gay/Homosexual/PLU	325 (73.7)	194 (72.9)	131 (74.9)	

Heterosexual/straight	27 (6.1)	9 (3.4)	18 (10.3)	
Queer/gender queer †	7 (1.6)	5 (1.9)	2 (1.1)	
Sexual behaviour				
Engaging in anal intercourse without condom with male sexual partner in the past 6 months				
Yes	213 (48.3)	135 (50.8)	78 (44.6)	0.207
No	228 (51.7)	131 (49.2)	97 (55.4)	
Symptoms related to sexually transmitted infections in the last year				
Yes	111 (25.2)	82 (30.8)	29 (16.6)	0.001
No	330 (74.8)	184 (69.2)	146 (83.4)	
History of sexually transmitted infections in the last year				
Yes	118 (26.8)	86 (32.3)	32 (18.3)	0.001
No	323 (73.2)	180 (67.7)	143 (81.7)	
Knowledge related to HPV and HPV infection				
Total knowledge score				
Low score (0–7)	209 (47.4)	97 (36.5)	112 (64.0)	p<0.0001
High score (8–13)	232 (52.6)	169 (63.5)	63 (36.0)	
Attitudes towards HPV infection				
Level of chance of getting HPV infection				
Very high/High	158 (35.8)	126 (47.4)	32 (18.3)	p<0.0001
Low/Very low	283 (64.2)	140 (52.6)	143 (81.7)	
Level of worriedness about getting disease from HPV infection				
Very high/High	250 (56.7)	189 (71.1)	61 (34.9)	p<0.0001
Low/Very low	191 (43.3)	77 (28.9)	114 (65.1)	
The chance of HPV infection seems small to me				
Strongly agree/Agree	128 (29.0)	67 (25.2)	61 (34.9)	0.032
Disagree/Strongly disagree	313 (71.0)	199 (74.8)	114 (65.1)	
HPV infection is not important enough to get vaccinated against				
Strongly agree/Agree	46 (10.4)	23 (8.6)	23 (13.1)	0.152
Disagree/Strongly disagree	395 (89.6)	243 (91.4)	152 (86.9)	

The diseases I may get due to HPV infections are not serious				
Strongly agree/Agree	14 (3.2)	4 (1.5)	10 (5.7)	0.023
Disagree/Strongly disagree	427 (96.8)	262 (98.5)	165 (94.3)	
HPV infection is a bigger problem for women than it is for men				
Strongly agree/Agree	34 (7.7)	17 (6.4)	17 (9.7)	0.207
Disagree/Strongly disagree	407 (92.3)	249 (93.6)	158 (90.3)	
Attitudes towards HPV vaccination				
Getting vaccinated against HPV has more drawbacks than benefits				
Strongly agree/Agree	22 (5.0)	10 (3.8)	12 (6.9)	0.180
Disagree/Strongly disagree	419 (95.0)	256 (96.2)	163 (93.1)	
I am against vaccination in general				
Strongly agree/Agree	15 (3.4)	7 (2.6)	8 (4.6)	0.293
Disagree/Strongly disagree	426 (96.6)	259 (97.4)	167 (95.4)	
I do not have enough information about HPV vaccine to decide on vaccination				
Strongly agree/Agree	305 (69.2)	204 (76.7)	101 (57.7)	p<0.0001
Disagree/Strongly disagree	136 (30.8)	62 (23.3)	74 (42.3)	
The long-term potential side effects of HPV vaccination remain unclear				
Strongly agree/Agree	312 (70.7)	210 (78.9)	102 (58.3)	p<0.0001
Disagree/Strongly disagree	129 (29.3)	56 (21.1)	73 (41.7)	
It is embarrassing for men to take up HPV vaccines				
Strongly agree/Agree	120 (27.2)	70 (26.3)	50 (28.6)	0.662
Disagree/Strongly disagree	321 (72.8)	196 (73.7)	125 (71.4)	
It is troublesome to take up HPV vaccines				
Strongly agree/Agree	152 (34.5)	98 (36.8)	54 (30.9)	0.219
Disagree/Strongly disagree	289 (65.5)	168 (63.2)	121 (69.1)	
Taking up the HPV vaccine may be a sign of promiscuity				
Strongly agree/Agree	144 (32.7)	96 (36.1)	48 (27.4)	0.062
Disagree/Strongly disagree	297 (67.3)	170 (63.9)	127 (72.6)	

I find HPV vaccination a sensitive topic because it has to do with sexual activity				
Strongly agree/Agree	210 (47.6)	143 (53.8)	67 (38.3)	0.002
Disagree/Strongly disagree	231 (52.4)	123 (46.2)	108 (61.7)	
I am confident that I could take up HPV vaccines if I wanted to				
Strongly agree/Agree	349 (79.1)	244 (91.7)	105 (60.0)	p<0.0001
Disagree/Strongly disagree	92 (20.9)	22 (8.3)	70 (40.0)	
I will take an HPV vaccine if a doctor recommends me to take up HPV vaccines				
Strongly agree/Agree	364 (82.5)	252 (94.7)	112 (64.0)	p<0.0001
Disagree/Strongly disagree	77 (17.5)	14 (5.3)	63 (36.0)	
I will take an HPV vaccine if my friends recommend me to take up HPV vaccines				
Strongly agree/Agree	329 (74.6)	233 (87.6)	96 (54.9)	p<0.0001
Disagree/Strongly disagree	112 (25.4)	33 (12.4)	79 (45.1)	
Stigma				
I anticipate stigma in healthcare settings surrounding HPV vaccination for gay and bisexual men				
Yes	334 (75.7)	199 (74.8)	135 (77.1)	0.650
No	107 (24.3)	67 (25.2)	40 (22.9)	
